# Supplementary material for: TALE factors use two distinct functional modes to control an essential zebrafish gene expression program
Source: eLife. 2018 Jun 18;7:e36144. doi: 10.7554/eLife.36144 (PMC6023610; doi:10.7554/eLife.36144)
Supplement: Supplementary file 1. — Related to Figure 2. Prep ChIP-seq experiments were performed on 3.5hpf and 12hpf zebrafish embryos. Biological replicates (n = 2) for each condition show a high degree of reproducibility as shown by the percent of peaks found in both replicates (replicate overlap). Peaks with Fold Enrichment (FE) ≥ 10 were used for subsequent analysis. [file elife-36144-supp1.docx]

Supplementary File 1. ChIP-seq statistics. Related to Figure 2.

| Target | Stage (hpf) | Replicates | Replicate overlap | FE≥10 peaks |
| --- | --- | --- | --- | --- |
| Prep | 3.5 | 2 | 95.6% | 13,328 |
| Prep | 12 | 2 | 98.8% | 24,199 |

Prep ChIP-seq experiments were performed on 3.5hpf and 12hpf zebrafish embryos. Biological replicates (n=2) for each condition show a high degree of reproducibility as shown by the percent of peaks found in both replicates (replicate overlap). Peaks with Fold Enrichment (FE)≥10 were used for subsequent analysis.
